# Biologically active pigment and ShlA cytolysin of *Serratia marcescens* induce autophagy in a human ocular surface cell line

**DOI:** 10.1186/s12886-020-01387-z

**Published:** 2020-03-26

**Authors:** Kimberly M. Brothers, Nicholas A. Stella, Robert M. Q. Shanks

**Affiliations:** The Charles T. Campbell Ophthalmic Microbiology Laboratory, Department of Ophthalmology, University of Pittsburgh School of Medicine, EEI 1020, 203 Lothrop Street, Pittsburgh, Pennsylvania 15213 USA

**Keywords:** Autophagy, Bacteria, Keratitis, Ocular surface, Epithelium, Cornea, Prodigiosin, Cytolysin, Flagella, Fimbriae

## Abstract

**Background:**

The cellular process of autophagy is essential for maintaining the health of ocular tissue. Dysregulation of autophagy is associated with several ocular diseases including keratoconus and macular degeneration. It is known that autophagy can be used to respond to microbial infections and that certain microbes can exploit the autophagic process to their benefit. In this study, a genetic approach was used to identify surface-associated and secreted products generated by the opportunistic pathogen *Serratia marcescens* involved in activation of autophagy.

**Methods:**

A recombinant human corneal limbal epithelial cell line expressing a LC3-GFP fusion protein was challenged with normalized secretomes from wild-type and mutant *S. marcescens* derivatives. LC3-GFP fluorescence patterns were used to assess the ability of wild-type and mutant bacteria to influence autophagy. Purified prodigiosin was obtained from stationary phase bacteria and used to challenge ocular cells.

**Results:**

Mutations in the global regulators *eepR* and *gumB* genes highly reduced the ability of the bacteria to activate autophagy in corneal cells. This effect was further narrowed down to the secreted cytolysin ShlA and the biologically active pigment prodigiosin. Purified prodigiosin and ShlA from *Escherichia coli* further supported the role of these factors in activating autophagy in human corneal cells. Additional genetic data indicate a role for flagellin and type I pili, but not the nuclease, S-layer protein, or serratamolide biosurfactant in activation of autophagy.

**Conclusions:**

This work identifies specific bacterial components that activate autophagy and give insight into potential host-pathogen interactions or compounds that can be used to therapeutically manipulate autophagy.

## Background

Cells use autophagy to eliminate waste products such as damaged organelles and proteins in order to enhance survival during periods of starvation. Autophagy dysregulation has been linked to many diseases including those of the eye [1–7]. Therefore therapeutic control of autophagy has been suggested for treatment of cancer, metabolic diseases, neurodegenerative disorders, for management of cardiovascular aging, and even for treatment of corneal infections [8–10].

The role of autophagy in the cornea is less well understood, but it is clear that autophagy plays a role in HSV-1 infection and keratoconus [3, 5, 11]. A recent study measured activation of autophagy in mouse corneas following infection with the fungus *Aspergillus fumigatus* and positively correlated autophagy with the severity of infectious pathology [12]. Similarly, data from a study using the bacterium *Pseudomonas aeruginosa*, suggest that it benefits from activating autophagy as a means of escaping extracellular killing in macrophages [13]. However, in general, activation of autophagy is thought to protect cells from microbial infection [14, 15]. It is known that a few bacterial proteins such as TlpE from *P. aeruginosa*, bacterial macrolide, rapamycin, TLR-ligands, and proinflammatory cytokines can activate autophagy [15–17], but knowledge of the scope of infectious components that activate autophagy is limited [15].

Our previous work has demonstrated that sterile culture filtrates (secretomes) of a number of ocular pathogens can activate autophagy in a human corneal limbal epithelial cell line [18], impede cell migration and wound closure [19], and cause cellular death in a bacterial-strain dependent manner [20, 21]. These included secretomes gram positive bacteria such as *Staphylococcus aureus* and gram negative bacteria including *Serratia marcescens* [18]. The secreted or shed bacterial components detected by the corneal cells that activate autophagy were not determined. In this study we took advantage of our collection of *S. marcescens* defined mutants to identify bacterial factors that induce autophagy in corneal cells.

## Methods

### Analysis of autophagy induced by keratitis isolates

Bacterial stocks (Table 1) were stored at − 80 °C and single colonies were obtained on lysogeny broth (LB) agar. Colonies were grown in LB at 30 °C for ~ 18 h with aeration on tissue culture rollers. Where noted, bacteria were grown with L-arabinose at 1 mM for controlled expression of genes. Secretomes were prepared by normalizing overnight cultures to OD_600_ = 2.0, removal of bacteria by centrifugation at 14,000 rpm and filtration through a 0.22 μm filter (Millex PVDF). Normalized secretomes were added to HCLE cells at a ratio of 500 μl per 1 ml of tissue culture medium (KSFM) and incubated at 37 °C + 5% CO_2_ for 3 h. In some cases secretomes were further diluted 2-fold (OD_600_ = 1.0) due to excessive cytotoxicity as noted in the text. The autophagy inhibitor 3-methyladenine (3MA) was added to culture media at 5 mM, one hour prior to challenge with secretomes as previously described [18, 31].

**Table 1:** Bacteria and plasmids used in this study

Strain or plasmid	Description	Reference or source
Top10	*E. coli* laboratory strain	ThermoFisher
PIC3611	*Serratia marcescens* wild-type strain	Presque Isle Cultures
K904	*S. marcescens* keratitis isolate	[22]
CMS1722	PIC3611 with pMQ262 (L-arabinose inducible *pig*)	[23]
CMS2096	PIC3611 ∆*pigA*	[24]
CMS2097	PIC3611 ∆*eepR*	[25]
CMS2229	K904 *pigD*::tn	This study
CMS2232	K904 *swrW*::tn	[26]
CMS2904	K904 ∆*eepR*	[25]
CMS3559	K904 *nucA*::tn	This study
CMS3900	K904 *fliC*::pMQ192	[27]
CMS4001	K904 ∆*gumB*	[28]
CMS4225	K904 *fimC*::pMQ167	[27]
CMS4236	K904 ∆*shlB*	[20]
CMS4334	K904 *shlA*::tn	[20]
CMS4413	K904 ∆*slaA*	[29]
CMS4773	K904 ∆*shlB pigE*::tn	This study
Plasmids		
pMQ125	expression vector with L-arabinose inducible promoter	[30]
pMQ492	pMQ125 with *shlBA* operon from *S. marcescens*	[20]

To analyze autophagy, LC3-GFP HCLE cells [18] were imaged with 60X magnification on an Olympus IX-81 inverted confocal microscope with Fluoview imaging software. The LC3-GFP cells were generated by lentiviral transduction of the human corneal limbal epithelial cell line from Ilene Gipson [32]. Image J (NIH) was used to quantify images without any image adjustment. Autophagy levels were quantified following recommendations of Klionsky et al. [33] in which the standard deviation of fluorescent pixel intensity of a cell is divided by its mean pixel intensity of the cell. Two to three fields per treatment condition were imaged. The experiment was repeated on at least two different days and at least 50 cells were analyzed per group. The data was averaged from all fields taken per experiment and graphed using GraphPad prism. One way ANOVA with Tukey’s post hoc analysis was used to determine statistical significance at *P* < 0.05 unless otherwise stated.

### Generation of new mutants strains

Additional mutants (Table 1) were generated by transposon mutagenesis using the pSC189 mariner transposon delivery system as previously described [34, 35]. Transposons were mapped using the method of Chiang, et al. [34] To identify nuclease defective mutants, libraries of mutants were transferred from 96-well plates to DNase detection agar (BD Difco) and plates were screened for loss of nuclease zones after 16–20 h of incubation at 30 °C around individual colonies. The transposon was mapped to 89 bp upstream of the *nucA* open reading frame and results in an almost complete loss of secreted nuclease activity (data not shown). Mutations in the prodigiosin biosynthetic operon were obtained by visually screening mutant libraries of strain K904 and K904 ∆*shlB* for loss of pigment. Transposon insertions were mapped to base pair 2451 in the K904 strain background and to base pair 1075 of *pigE* in the ∆*shlB* strain background.

### Purification of prodigiosin

Wild-type strain PIC3611 and an isogenic ∆*pigA* strain were grown overnight in LB broth with aeration. The ∆*pigA* mutant does not make prodigiosin and served as a negative control. Bacteria were adjusted to OD_600_ = 4, aliquots (5 ml) were pelleted by centrifugation (7000 RPM for 10 min), and supernatants were removed. To extract prodigiosin, bacterial cells were suspended in 100 μl of acidified ethanol (2 ml of 2 M HCl in 98 ml of 95% ethanol) and incubated for one hour with periodic vortexing. Samples were further purified with hydroxyapatite resin. Columns were packed with hydroxyapatite resin (BioRad #16260), equilibrated with acidified ethanol, and samples were run through the columns with acidified ethanol. The mock purification sample from the ∆*pigA* culture was collected at the same time as the prodigiosin fraction from the wild type was collected. Samples were air-dried and prodigiosin concentration was determined using a standard curve of absorbance at 534 nm using commercial prodigiosin as a standard (Sigma). The same volume of prodigiosin was added from the wild type and ∆*pigA*-derived samples.

## Results

### S. marcescens secretome induction of autophagy is inhibited by 3-methyladenine

Our previous study showed that a subset of ocular bacterial pathogens induced autophagy in a corneal cell line, and that among the strongest induction was observed with *S. marcescens* [18]. In this study, we set out to determine which components of *S. marcescens* using wild-type and genetically manipulated strains induced formation of LC3-GFP puncta. We used two *S. marcescens* strains: strain PIC3611 from Presque Isle Culture collection, a laboratory strain that is likely from an environmental source (biotype TCT), and K904, a contact lens associated keratitis isolate (biotype A6a). Two strains were used to determine whether phenotypes were associated with one particular strain or a more general phenomenon.

**Fig. 1:**
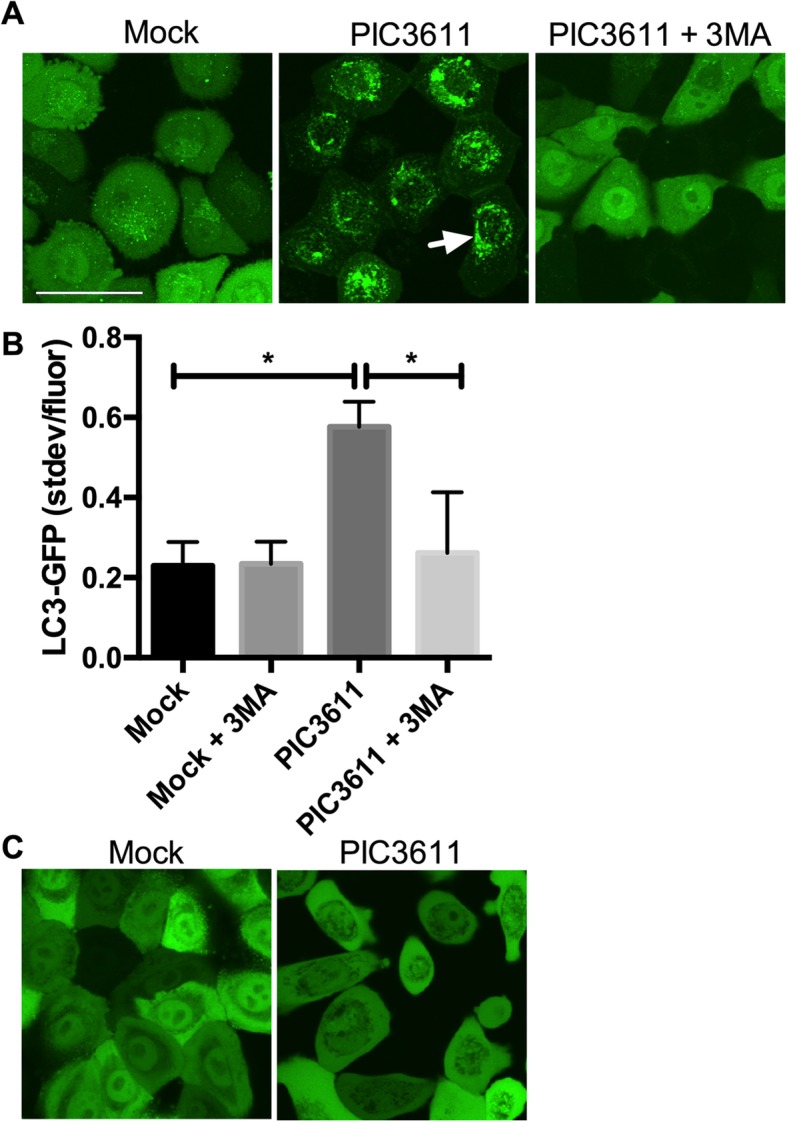
Induction of autophagy in a corneal cell line by *S. marcescens*. **a**. Activation of autophagy by secretome of wild-type strain PIC3611 that can be reversed by addition of autophagy inhibitor 3-methyladenine (3MA). Representative confocal images of HCLE LC3-GFP cells exposed to LB medium (mock), secretome from PIC3611, or secretome with autophagy inhibitor. White arrow indicates LC3-GFP puncta and scale bar = 50 μm. **b**. Autophagy levels were measured using LC3-GFP patterns. Averages and standard deviations are shown, *n* > 50 cells per group. Asterisks denote significant differences using ANOVA with Tukey’s post-test (*p* < 0.05). **c**. Representative confocal images of HCLE-GFP cells showing no puncta after being exposed to PIC3611 secretomes

Figure 1 demonstrates activation of autophagy following exposure of HCLE LC3-GFP cells to normalized filtered supernatants (secretomes) from strain PIC3611 Fig. 1a, b). The formation of LC3-GFP puncta indicate cells with activated autophagy and can be used in quantification of autophagy [33]. Autophagy stimulation by PIC3611 secretomes could be prevented using autophagy inhibitor 3-methyladenine (3MA), supporting that the observed LC3-GFP phenotype is autophagy dependent (Fig. 1). The same trend was previously shown for strain K904 and with the use of autophagy inhibitor baflomycin [18]. As an additional control to show that LC3-GFP puncta are not an artifact of fluorescent bacterial components, an HCLE-GFP cell line with no LC3 fusion was used [18]. Following PIC3611 supernatant treatment, no fluorescent focus formation was observed from the GFP control cell line (Fig. 1c).

### S. marcescens secondary metabolite regulators are necessary for activation of autophagy

**Fig. 2:**
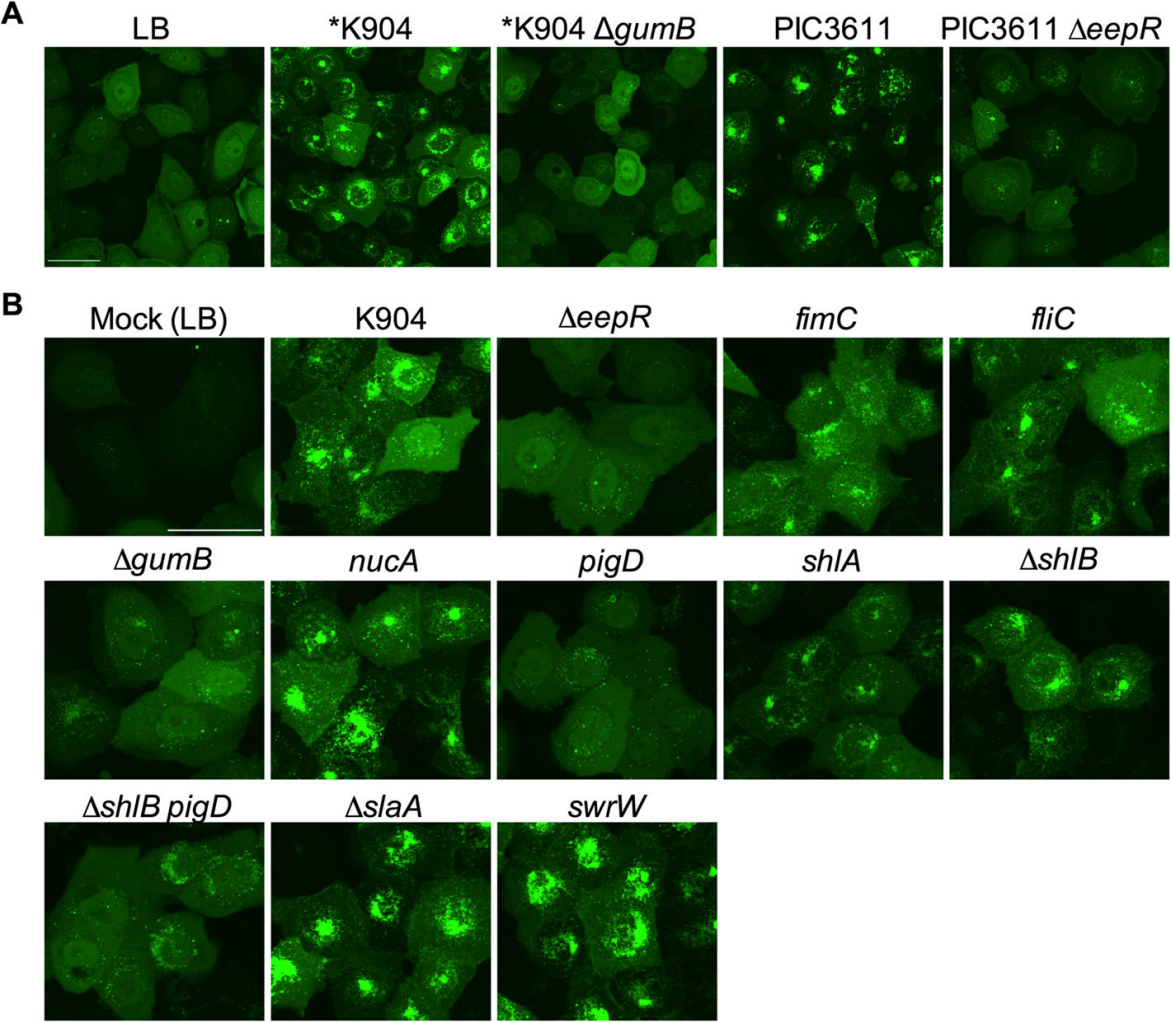
**a**. Activation of autophagy by *S. marcescens* secretomes. Images of LC3-GFP puncta in HCLE cells treated with secretomes. OD_600_ = *0.5 secretomes used in the shown experiment. Scale bar = 50 μm. **b**. Activation of autophagy by secretomes from various isogenic mutants in the K904 strain background. Scale bar = 50 μm

The component(s) of the secretome responsible for autophagy induction is unknown. Our previous study demonstrated that secretomes could be heated at 95 °C and still induce autophagy [18]. Well-defined *S. marcescens* strains with deletion mutations in the *eepR* and *gumB* genes were tested for loss of autophagy induction because these genes have a conserved and broad impact on *S. marcescens* biology [20, 24, 25, 28, 36]. The EepR and GumB genes regulate expression of multiple secreted factors including heat-stable secondary metabolites such as the biologically active pigment prodigiosin and the hemolytic and antimicrobial biosurfactant serratamolide. Both the *eepR* and *gumB* mutants were defective in activation of autophagy (Fig. 2a).

**Fig. 3:**
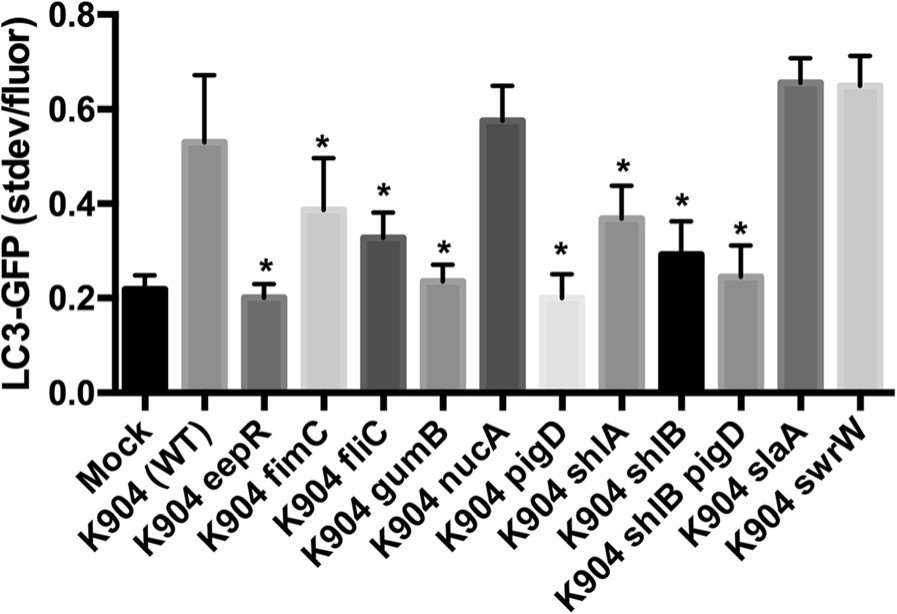
*S. marcescens* mutants unable to secrete prodigiosin pigment or ShlA cytolysin are defective in inducing autophagy. Activation of autophagy by wild-type secretome (K904) and isogenic mutants or mock (LB medium). Asterisks denote significant differences from wild-type strain K904 using ANOVA with Tukey’s post-test (*p* < 0.05). Averages and standard deviations are shown, *n* > 50 cells per group

Additionally, secretomes from strains with mutations in a variety of surface or secreted proteins regulated by EepR and/or GumB were individually tested in the K904 strain background (Figs. 2b and 3). These include the type 1 pilus gene, *fimC*, the flagellin gene, *fliC*, the secreted nuclease gene, *nucA*, the prodigiosin biosynthetic gene, *pigD*, the cytolysin, *shlA*, the outer membrane cytolysin transporter gene *shlB*, the S-layer protein gene, *slaA*, and the serratamolide biosynthetic gene, *swrW*.

Mutant analysis suggested that the *eepR* and *gumB* mutants share a defect in autophagy induction with a subset of the mutants (Figs. 2b and 3), most notably *pigD*, which codes for a gene involved in prodigiosin pigment biosynthesis [37]. Both *eepR* and *gumB* are severely defective in prodigiosin production due to reduced transcription of the prodigiosin biosynthetic genes, implicating prodigiosin as a stimulatory factor [25, 28]. Secretomes from mutants unable to make type 1 pili, flagella, and cytolysin / cytolysin transporter (*shlA* and *shlB*, respectively) were also defective, but to a lesser extent then the pigment mutants.

### Prodigiosin is necessary and sufficient to induce autophagy in a corneal cell line

**Fig. 4:**
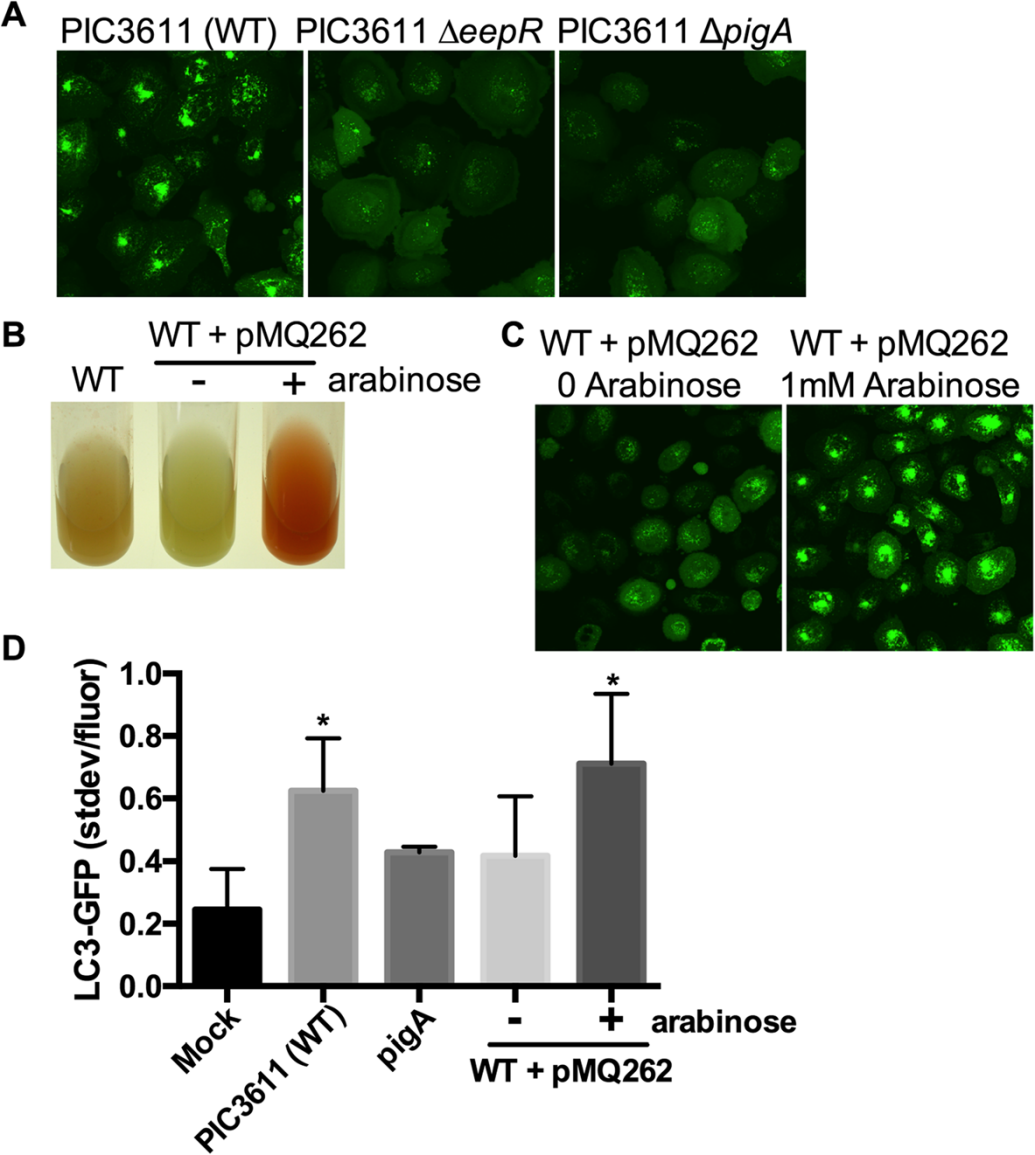
Prodigiosin biosynthetic genes are necessary for maximal autophagy induction by *S. marcescens* strain PIC3611. **a**. The *pigA* pigment biosynthetic gene is required for strain PIC3611 to induce autophagy and is defective to a similar level as the *eepR* mutant. **b**. Image of cultures of *S. marcescens* strain PIC3611 or PIC3611 with pMQ262 grown for 18 h without or with L-arabinose (1 mM), which activates prodigiosin biosynthetic gene transcription. Prodigiosin pigment is visible as orange to red coloration. **c**. Confocal images of LC3-GFP puncta activated by WT (PIC3611) + pMQ262 secretomes that had been grown with arabinose. **d**. Analysis of autophagy demonstrates a requirement for prodigiosin biosynthetic genes. Asterisks denote significant differences from the PIC3611 ∆*pigA* mutant using ANOVA with Tukey’s post-test (*p* < 0.05). Averages and standard deviations are shown, *n* > 50 cells per group

Given that the prodigiosin defective mutants, *eepR*, *gumB*, and *pigD* were defective in inducing autophagy whether they were from the PIC3611 (Fig. 2a) or K904 background (Figs. 2a, b and 3), we analyzed whether prodigiosin played a role in inducing autophagy in the HCLE LC3-GFP cell line in greater depth. First, because the *eepR* mutation confers pleiotropic effects, we compared the wild-type strain PIC3611 with an isogenic mutant unable to make PigA, which is required for prodigiosin biosynthesis. Similar to the *eepR* mutant, when only pigment biosynthesis was ablated through deletion of the *pigA* gene, strain PIC3611 was unable to induce autophagy (Fig. 4a).

We have previously described the use of plasmid pMQ262, which replaces the normal pigment biosynthetic promoter with an arabinose inducible promoter [26, 38], such that prodigiosin pigment biosynthesis is dependent upon arabinose in the growth medium (Fig. 4b). When secretomes from strain PIC3611 with pMQ262 were used to challenge HCLE LC3-GFP cells, the ability to activate autophagy correlated with arabinose induction of pigment production (Fig. 4c). Arabinose, itself, did not induce autophagy (data not shown). These data suggest that prodigiosin is necessary for *S. marcescens* secretomes to fully activate autophagy in corneal cells.

**Fig. 5:**
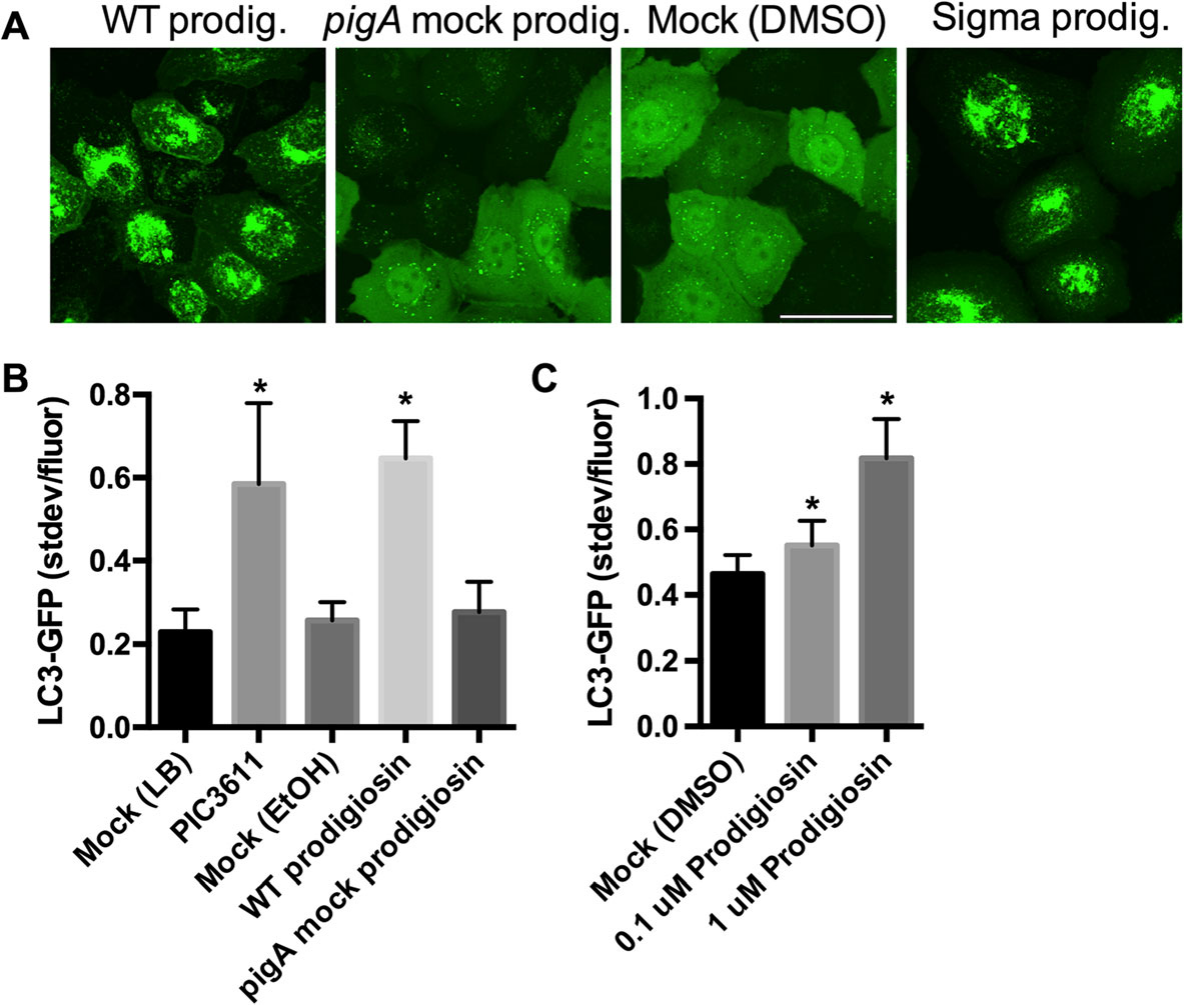
Prodigiosin is sufficient to induce autophagy in the corneal HCLE LC3-GFP cell line. **a**. Confocal images of HCLE LC3-GFP cells challenged with prodigiosin from wild-type PIC3611, the ∆*pigA* mutant, or from a commercial source (1 mM). **b**. Autophagy activation by WT PIC3611 secretome and prodigiosin purified from the WT but not isogenic ∆*pigA* mutant. **c**. Commercially available prodigiosin induces autophagy. Asterisks denote significant differences from Mock groups using ANOVA with Tukey’s post-test (*p* < 0.05). Averages and standard deviations are shown, *n* > 50 cells per group

To test whether prodigiosin was sufficient for activation of autophagy in HCLE LC3-GFP cell line, we purified prodigiosin from strain PIC3611 and mock purified it from the isogenic ∆*pigA* mutant, and tested these for activation of LC3-GFP puncta formation (Fig. 5a-b). Prodigiosin from PIC3611 (0.9 μM) was able to activate autophagy. The negative control mock purified prodigiosin from the ∆*pigA* mutant was unable to activate autophagy when added at the same volume. Similarly, commercially available prodigiosin could activate autophagy in a dose dependent manner (Fig. 5a, c).

### Analysis of the ShlA cytolysin in inducing autophagy from corneal cells

Data from genetic analysis above suggested that the pore forming cytolysin, ShlA, contributes to autophagy induction (Figs. 2b and 3). The ShlB protein is necessary for secretion of ShlA, such that *shlB* mutants do not secrete ShlA [39]. Consistently, the tested *shlB* mutant behaved similarly to the *shlA* mutant (Fig. 2b, and Fig. 3). We generated a double mutant that is unable to make prodigiosin or secrete ShlA (∆*shlB pigD*), and this was indistinguishable from the *pigD* mutant, but had a trend to lower levels of autophagy induction compared to the ∆*shlB* levels.

**Fig. 6:**
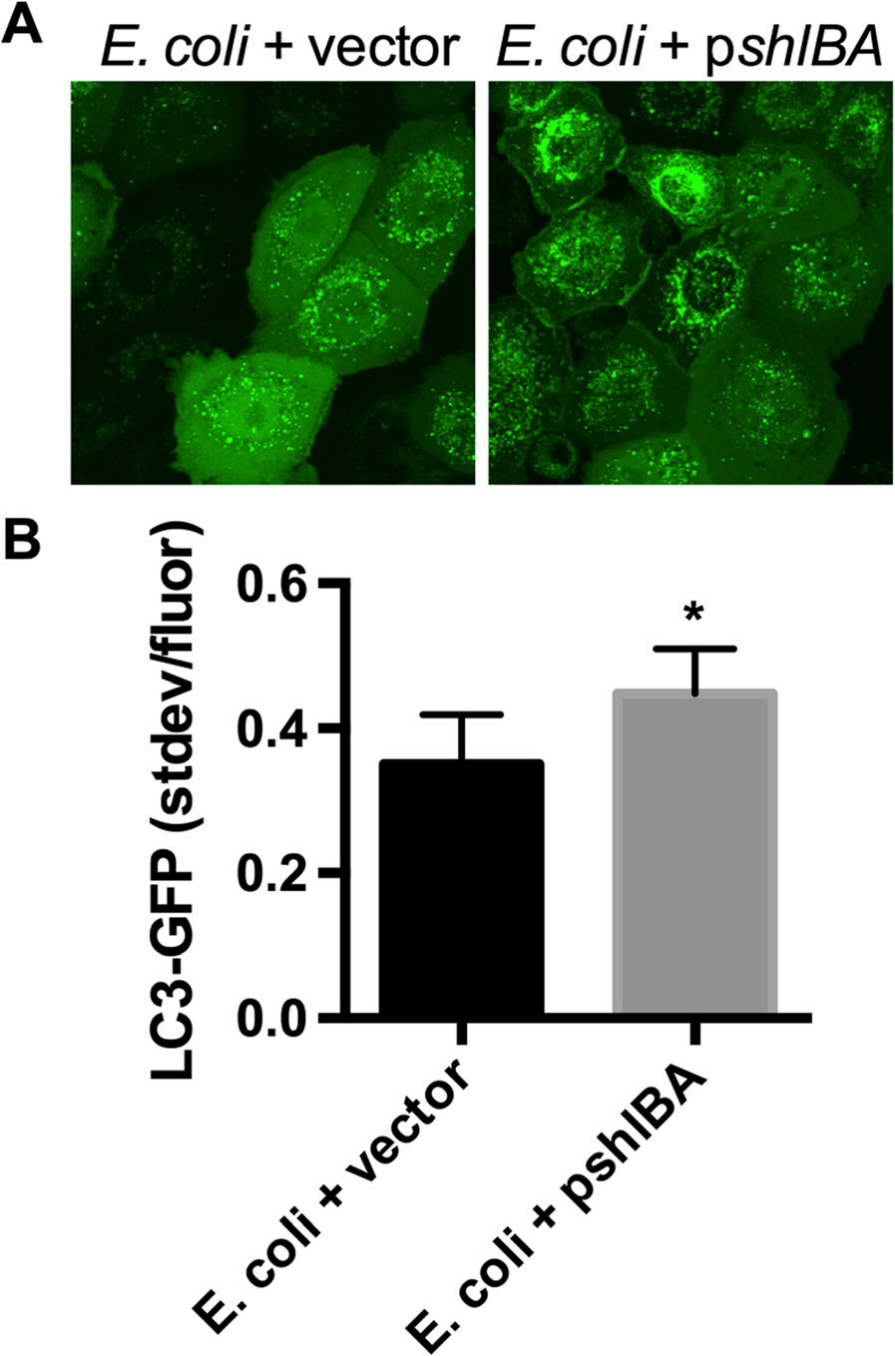
Expression of *S. marcescens* cytolysin operon in *E. coli* elevates its ability to activate autophagy by corneal cells. Confocal images (**a**) and LC3-GFP quantitation (**b**) of *E. coli* laboratory strain Top10 with a vector control (pMQ125) or a plasmid with *shlBA* (pMQ492). Averages and standard deviations are shown, *n* > 50 cells per group. Asterisk indicates significant difference by Student’s T-test (*p* < 0.05)

Our previous study detected moderate activation of autophagy by a clinical keratitis isolate of *E. coli* in HCLE-GFP cells [18] and that *E. coli* with a *shlBA* plasmid (pMQ492) is able to secrete functional ShlA cytolysin [20]. Here, we observed that ectopic expression of the *S. marcescens shlBA* operon increased the ability of *E. coli* secretomes to induce autophagy in HCLE LC3-GFP cells (Fig. 6). Together, these results indicate a role for the ShlA cytolysin in activation of autophagy.

## Discussion

Several studies have explored the role of ocular autophagy with HSV-1, *Toxoplasma gondii*, and fungal spp. [2, 3, 5] However, the role of ocular autophagy in response to bacterial pathogens remains poorly understood.

This study demonstrated that two strains of *S. marcescens* from different biotypes were capable of activating autophagy in a corneal cell line and identified bacterial factors capable of activating autophagy. Mutations in two different genes that confer major pleiotropic effects on *S. marcescens* behavior, *eepR* and *gumB*, prevented bacterial activation of autophagy. The *eepR* gene is a transcription factor that is required for wild-type levels bacterial proliferation in a rabbit keratitis model as well as positive regulation of secondary metabolites such as prodigiosin and serratamolide [24, 25]. The *gumB* gene codes for a stress response signal transmitting protein that positively regulates prodigiosin and serratamolide, and is necessary for production of the ShlA, ShlB, and flagellin [20, 28]. We therefore tested individual genes controlled by EepR and GumB and identified several bacterial factors that activate autophagy.

Our genetic and biochemical results indicate that prodigiosin can activate autophagy in the tested human corneal cell line. Prodigiosin, 2-methyl-3-pentyl-6-methoxyprodiginine, is thought to contribute to bacterial competition, and has antitumor capabilities [37, 40, 41]. Furthermore, prodigiosin was recently shown to activate autophagic cell death in a variety of cancer cell lines and to reduce tumor proliferation in mouse tracheas [42–49]. Many clinical isolates of *S. marcescens* do not synthesize prodigiosin [50], and perhaps this benefits them by reducing activation of the host’s innate immune response.

Beyond prodigiosin, data from this study implicated the ShlA cytolysin in activation of autophagy in corneal cells. Similarly, in an elegant study by the Véscovi group, the pore forming cytolysin ShlA was demonstrated to induce autophagy in Chinese hamster ovary (CHO) cells [51].

In contrast to our work that suggested a role for flagellin as an autophagy inducer, Di Venanzio showed that *S. marcescens* with mutations in *fliA* and *flhD*, which should be defective in flagella production, were able to activate autophagy in CHO cells [51]. These differences may be due to the specific bacterial strain background or use of CHO cells versus corneal cells. However, consistent with our finding, data from a recent papers using *Salmonella*, implicated flagellin as an activator of autophagy in zebrafish and murine RAW cells [52, 53]. To our knowledge there is no previous information on fimbriae / type I pili in activation of autophagy. It is also formally possible that some of the increase in LC3-GFP puncta results from a reduction in autophagic flux leading to the increase in overall autophagosomes. The impact of these bacterial factors on autophagic flux will be tested in subsequent studies.

## Conclusions

We have identified *S. marcescens* activators of autophagy. Whereas prodigiosin and ShlA from *S. marcescens* have been previously implicated in activating autophagy, this report is the first to demonstrate this with ocular derived cells. The ability of flagellin and fimbria to induce autophagy will need to be further validated using biochemical means, but this report identifies these bacterial factors as potential microbial mediators of autophagy in corneal cells. Since *S. marcescens* is most commonly associated with the eye as a contact lens associated pathogen, it is possible that corneal cells prime themselves for microbial infection through sensing prodigiosin, flagellin, fimbriae, and ShlA toxins.

## Data Availability

The datasets used during this current study are available from the corresponding author on reasonable request.

## Abbreviations

3MA: 3-methyladenine, autophagy inhibitor
ANOVA: Analysis of variance, statistical analysis
CHO: Chinese hamster ovary, cell line
GFP: Green fluorescent protein
HCLE: Human corneal limbal epithelial, cell line
RPM: Rotations per minute
